# Two New Polyphenol Oxidase Genes of Tea Plant (*Camellia sinensis*) Respond Differentially to the Regurgitant of Tea Geometrid, *Ectropis obliqua*

**DOI:** 10.3390/ijms19082414

**Published:** 2018-08-14

**Authors:** Chen Huang, Jin Zhang, Xin Zhang, Yongchen Yu, Wenbo Bian, Zhongping Zeng, Xiaoling Sun, Xinghui Li

**Affiliations:** 1 Tea Research Institute, Chinese Academy of Agricultural Sciences, Hangzhou 310008, China; yellowchen92@163.com (C.H.); zhangjin7981@163.com (J.Z.); xinzhang@tricaas.com (X.Z.); w935369897@163.com (Y.Y.); bwbkg21@163.com (W.B.); 2 Tea Research Institute, College of Horticulture, Nanjing Agricultural University, Nanjing 210095, China; zpzeng@njau.edu.cn; 3 Key Laboratory of Tea Biology and Resources Utilization, Ministry of Agriculture, Hangzhou 310008, China

**Keywords:** polyphenol oxidase, *Camellia sinensis*, *Ectropis obliqua*, wounding, regurgitant

## Abstract

Polyphenol oxidases (PPOs) have been reported to play an important role in protecting plants from attacks by herbivores. Though PPO genes in other plants have been extensively studied, research on PPO genes in the tea plant (*Camellia sinensis*) is lacking. In particular, which members of the PPO gene family elicit the defense response of the tea plant are as yet unknown. Here, two new PPO genes, *CsPPO1* and *CsPPO2*, both of which had high identity with PPOs from other plants, were obtained from tea leaves. The full length of *CsPPO1* contained an open reading frame (ORF) of 1740 bp that encoded a protein of 579 amino acids, while *CsPPO2* contained an ORF of 1788 bp that encoded a protein of 595 amino acids. The deduced CsPPO1 and CsPPO2 proteins had calculated molecular masses of 64.6 and 65.9 kDa; the isoelectric points were 6.94 and 6.48, respectively. The expression products of recombinant CsPPO1 and CsPPO2 in *Escherichia coli* were about 91 and 92 kDa, respectively, but the recombinant proteins existed in the form of an inclusion body. Whereas *CsPPO1* is highly expressed in stems, *CsPPO2* is highly expressed in roots. Further results showed that the expression of *CsPPO1* and *CsPPO2* was wound- and *Ectropis obliqua*-induced, and that regurgitant, unlike treatment with wounding plus deionized water, significantly upregulated the transcriptional expression of *CsPPO2* but not of *CsPPO1*. The difference between regurgitant and wounding indicates that *CsPPO2* may play a more meaningful defensive role against *E. obliqua* than *CsPPO1*. Meanwhile, we found the active component(s) of the regurgitant elicited the expression of *CsPPO* may contain small molecules (under 3-kDa molecular weight). These conclusions advance the understanding of the biological function of two new PPO genes and show that one of these, *CsPPO2*, may be a promising gene for engineering tea plants that are resistant to *E. obliqua*.

## 1. Introduction

Plant polyphenol oxidases (PPOs), which are ubiquitous, dinuclear, copper-containing metalloproteins, contribute to the lignification, pigmentation, and, in higher plant species, defense against pathogens or herbivores [1,2,3,4,5]. PPOs utilize molecular oxygen to oxidize various phenolic precursors to their corresponding quinines [6], and these quinones are responsible for the enzymatic browning of many fruits, vegetables, and grains. Such browning often accompanies senescence, mechanical damage, and attack by pathogens or herbivores [7,8,9]. The negative effect of PPOs on the appearance and nutritional quality of products has prompted numerous ecological and molecular studies, as has the role of PPOs in plant defense against herbivores and pathogens [10,11,12,13,14,15]. For instance, an inverse correlation has been found between the performance of cotton bollworm (*Helicoverpa armigera* (Hübner)), beet armyworm (*Spodoptera exigua* (Hübner)), and PPO levels [12,16], and the antisense suppression of potato (*Solanum tuberosum* L.) *StPPO* has been shown to increase susceptibility, and the overexpression of PPO cDNAs has been demonstrated in tomato (*Lycopersicon esculentum* L.) to increase resistance to *Pseudomonas syringae* pv. *tomato* and to *Spodoptera litura* (Fabricius) [11,17,18]. In addition, *PPO* genes are frequently found to be differentially induced in response to injuries inflicted by wounding, pathogens, or herbivores from various plant species, and also to signaling molecules (jasmonic acid (JA), methyl jasmonate (MeJA), salicylic acid (SA), ethylene (ET)), suggesting that these genes have a defensive role [19,20,21,22,23].

To our knowledge, *PPO* gene families have been described in more than 26 plant species [22,24,25,26]: the *PPO* gene family of the tomato consists of seven members [27] and the family of *Salvia miltiorrhiza* comprises 19 *PPO* genes [22]. Until now, according to reports, only one full-length genomic DNA sequence of *PPO* has been cloned from *Camellia sinensis* cv. *Longjing 43* (EF635860.1), although the *PPO* gene family of *C. sinensis* (L.) O. Kuntzeis is thought to have from five to six members [28].

The tea plant, *C. sinensis*, is not only one of the world’s most important woody-plantation crops but is also valued as a source of secondary metabolic products, including phyto-oxylipins [29]. Tender tea buds and leaves are the raw material for commercial tea, one of the most popular nonalcoholic drinks worldwide [30]. Developing tea shoots and leaves may be damaged by numerous pests, such as the tea geometrid *Ectropis obliqua* (Prout), whose larvae seriously affect the yield and quality of tea [31], and the tea green leafhopper, *Empoasca onukii* (Matsuda). Herbivore-induced plant defenses are induced both by wounding, which is caused by the herbivore mouthparts involved in chewing/piercing, and by the elicitors/effectors that come from the insect’s oral or oviduct secretions [32,33]. Oral secretions (OS) are the key factors according to which the plant distinguishes between mechanical damage and herbivore feeding, and then responds, as different responses are elicited by different herbivore species [32,33,34,35,36,37]. Previously, we found that PPOs were an important antiherbivore factor in tea plants, defending them directly against *E. obliqua* larvae [4]. Though mechanical damage and JA treatment can upregulate PPO activity in tea leaves, both the infestation of the tea geometrids and wounding plus the regurgitant significantly suppressed wound-induced PPO activity, from which we inferred that *E. obliqua* larvae have evolved to be able to elude the tea plant’s defenses by inhibiting the production of PPOs [4]. Unfortunately, which genes are responsible for PPO activity induced or inhibited by the exogenous application of JA or the infestation of *E. obliqua* remains unknown.

To elucidate the *CsPPOs* responsible for the defense/coevolutionary response of the tea plant, we first isolated and characterized two new full-length cDNA sequences of *CsPPO* genes from *C. sinensis* cv. *Longjing 43*. Second, the phylogenetic relationship was analyzed by DNAMAN software. Third, we analyzed the transcriptional expression characteristics of these two genes in different tissues and in response to mechanical damage, *E. obliqua* infestation, and treatment involving mechanical damage plus regurgitant or exogenous application of JA. Finally, *CsPPO1* and *CsPPO2* were used as target genes to screen the active components of the regurgitant by detecting transcriptional expression levels of leaves that were treated with three separate compounds of the regurgitant. Our results will help clarify the interaction between tea plants and tea geometrids, and provide candidate defensive gene resources for breeding molecular resistance to insects in tea.

## 2. Results

### 2.1. cDNA Cloning and Sequence Analysis

**Figure 1:**
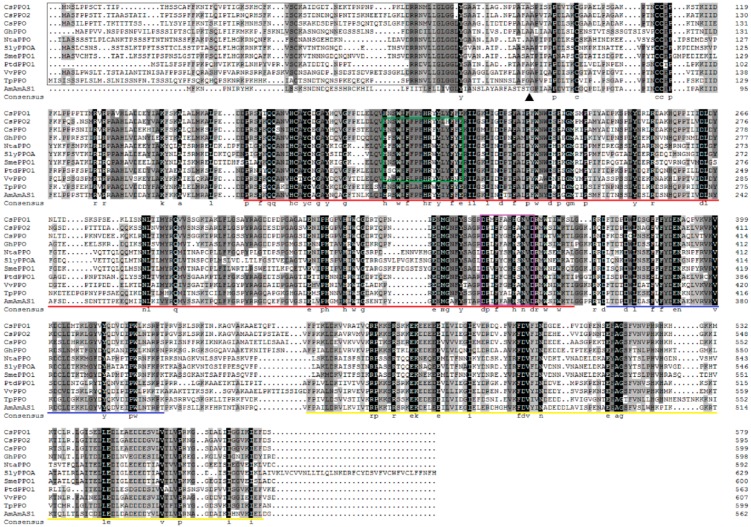
Amino acid sequence alignment of three polyphenol oxidases (PPOs) in *C. sinensis* and other plant PPOs. The region corresponding to the chloroplast transit peptide is boxed, and the thylakoid peptidase-processing site is indicated by ▲. The three domains: red underline, tyrosinase domain; blue underline, PPO1_DWL domain; yellow underline, PPO1_KFDV domain. The green box refers to the CuA binding site; the purple box to the CuB binding site.

The full length of the *CsPPO1* contained an ORF of 1740 bp that encoded a 579-amino acid residue, while the *CsPPO2* contained an ORF of 1788 bp that encoded a 595-amino acid residue. The deduced CsPPO1 and CsPPO2 proteins had calculated the molecular weight (Mw) of 64.6 kDa and 65.9 kDa, and the isoelectric points (pI) were 6.94 and 6.48, respectively. Compared to the published *CsPPO* (EF635860.1), the putative conserved domains of CsPPOs were predicted on a protein-blast website, and the results indicated that the *CsPPOs* we isolated are new genes encoding two new putative PPOs of the tea plant. The three cDNA sequences share 71–76% and 68–72% pairwise identity at the nucleic-acid and amino-acid levels, respectively (Table 1). The 80–90 residues of the derived amino-acid sequences of CsPPOs in the N-terminal region (Figure 1) show many typical features of a chloroplast transit peptide. The proteins had a conserved tyrosinase superfamily motif and two copper ion-binding sites. The PPO-DWL supermotif was the conserved domain of PPO that contained approximately 50 amino acids. The PPO-KFDV superfamily, whose function has not yet been studied, was the C-terminal domain of these oxidases (Figure 1). Interestingly, sequence analysis revealed only one copper-binding site in CsPPO1, TpPPO, and AmAS1, and two copper-binding sites in CsPPO2, CsPPO, GhPPO, NtaPPO, SlyPPOA, SmePPO1, PtdPPO1, and VvPPO (Figure 1).

### 2.2. The Phylogenetic Analysis

**Figure 2:**
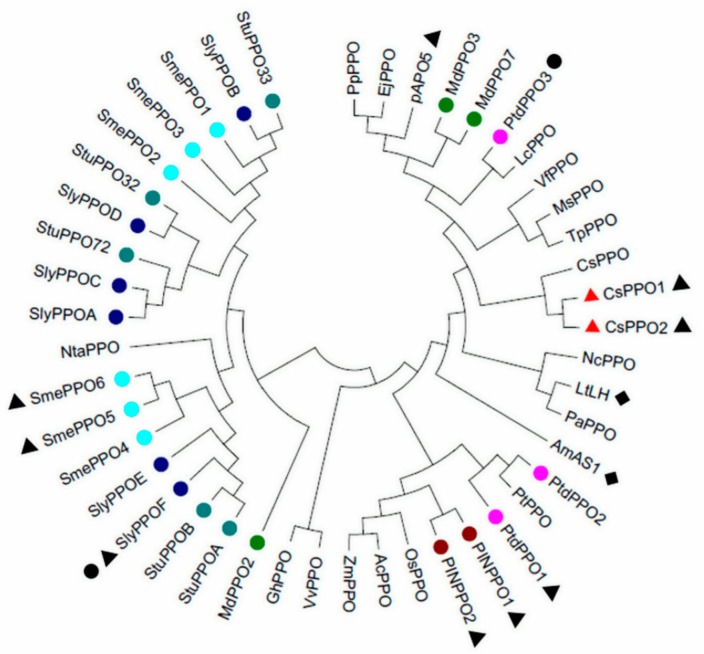
Phylogenetic analysis of PPO protein squences. The numbers on the tree branches represent bootstrap confidence values, as “Bootstrap” is 1000. The black triangles indicate wound-inducible; black dots indicate pathogen-inducible; black rhombuses indicate PPOs with biosynthetic functions. All sequence data may be found in GenBank, and differently colored bullet points represent different *PPO* gene families. *Malus* × *domestica* (*pAPO5*, P43309; *MdPPO2*, AAK56323; *MdPPO3*, BAA21676; and *MdPPO7*, BAA21677); *Pyrus pyrifolia* (*PpPPO*, AB056680); *Eriobotrya japonica* (*EjPPO*, AFO55217); *Vicia faba* (*VfPPO*, CAA77764.1); *Medicago sativa* (*MsPPO*, AAP33165.1); *Trifolium pretense* (*TpPPO*, AAK13244.1); *Populus trichocarpa* × *P. deltoids* (*PtdPPO1*, AAG21983.1; *PtdPPO2*, AAU12256.1; and *PtdPPO3*, AAU12257.1); *Litchi chinensis* (*LcPPO*, AEQ30073); *Phytolacca American* (*PaPPO*, D45386.1); *Larrea tridentata* (*LtLH*, AAQ67412); *Nelumbo nucifera* (*NcPPO*, ADP89908.1); *Vitis vinifera* (*VvPPO*, AB871370); *Camellia sinensis* (*CsPPO*, EF635860.1); *Populus trichocarpa* (*PtPPO*, AEH41424.1); *Ananas comosus* (*PINPPO1*, AAO16863.1; and *PINPPO2*, AAO16865.1); *Annona cherimola* (*AcPPO*, ABJ90144.1); *Oryza sativa* (*OsPPO*, DQ532396); *Zea mays* (*ZmPPO*, ACG28948.1); *Antirrhinum majus* (*AmAS1*, BAB20048.1); *Gossypium hirsutum* (*GhPPO*, AFC36521.1); *Solanum tuberosum* (*StuPPO32*, AAA85121.1; *StuPPO33*, AAA85122.1; *StuPPO72*, AAA85123.1; *StuPPOA*, AAA02877.1; and *StuPPOB*, AAA02879.1); Solanum lycopersicum (*SlyPPOA*, Q08303; *SlyPPOB*, Q08304; *SlyPPOC*, Q08305; *SlyPPOD*, Q08306; *SlyPPOE*, Q08307; and *SlyPPOF*, Q08296); *Solanum melongena* (*SmePPO*, ACR61399.1) and *Nicotiana tabacum* (*NtaPPO*, CAA73103.1).

On the basis of the alignment of the amino-acid sequences of several plant PPOs, a phylogenetic tree was generated using MEGA 6. The phylogenetic tree (Figure 2) showed that CsPPO1 and CsPPO2 were clustered on the same branch, suggesting that CsPPO1 and CsPPO2 have higher similarity to each other than to CsPPO. The CsPPOs are much closer to PPOs from red clover (*Trifolium pretense*), lotus flower (*Nelumbo nucifera*), and pokeweed (*Phytolacca americana*) than to PPOs from eggplant (*Solanum melongena*), tomato, and tobacco (*Nicotiana tabacum*). The PPO members of the nightshade family are clustered on the same branch, close to the NtasPPO. The rest of the PPO members from the same plant in the PPO family are relatively closer than others. Most of the PPO members from different plants are not inducible, and the inducible PPO members from the same plant are closer to each other than to the others.

### 2.3. Expression of the Recombinant Protein in E. coli

**Figure 3:**
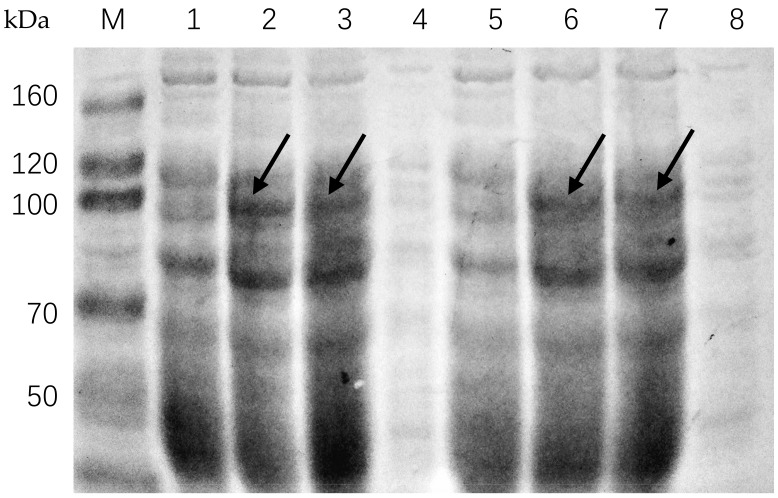
SDS-PAGE analysis of recombinant CsPPO1 and CsPPO2 proteins expressed in *E. coli*. The lanes M and 1−8 in the graph are as follows: M, protein molecular mass marker. 1, pGEX-4T-2/CsPPO1 without isopropyl-β-d-thiogalactopyranoside (IPTG) induction. 2, pGEX-4T-2/CsPPO1 with IPTG induction. 3, precipitation of pGEX-4T-2/CsPPO1 with IPTG induction after sonication. 4, supernatant of pGEX-4T-2/CsPPO1 with IPTG induction after sonication. 5, pGEX-4T-2/CsPPO2 without IPTG induction. 6, pGEX-4T-2/CsPPO2 with IPTG induction. 7, precipitation of pGEX-4T-2/CsPPO2 with IPTG induction after sonication. 8, supernatant of pGEX-4T-2/CsPPO2 with IPTG induction after sonication. The bands of pGEX-4T-2/CsPPO1 and pGEX-4T-2/CsPPO2 were pointed out by arrows in lanes 2, 3, 6, 7.

The expression vectors pGEX-4T-2/CsPPO1 and pGEX-4T-2/CsPPO2 were constructed and transferred into *E. coli* BL21 (DE3). *CsPPO1* and *CsPPO2* genes were highly expressed in *E. coli* cells. Results from a solubility analysis indicate that the recombinant proteins of pGEX-4T-2/CsPPO1 and pGEX-4T-2/CsPPO2 exist in the form of an inclusion body (Figure 3). The expression products of recombinant CsPPO1 and CsPPO2 in *E. coli* are about 91 and 92 kDa, respectively.

### 2.4. Expression of CsPPO1 and CsPPO2 in Different Tissues

**Figure 4:**
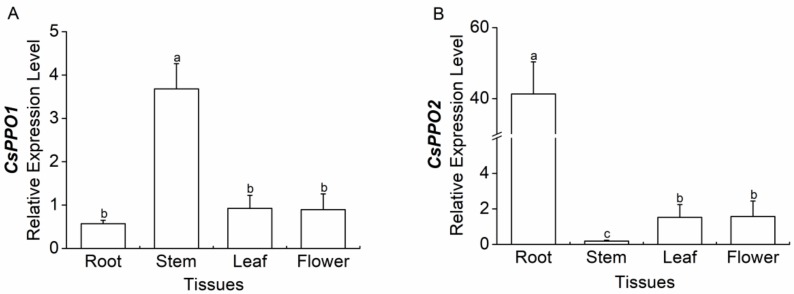
Mean levels of transcriptional expression (±SE) of (**A**) *CsPPO1* and (**B**) *CsPPO2* in tissues from the roots, stems, leaves, and flowers of *Camellia sinensis*. *CsGAPDH* was used as a reference gene. For each column, different letters indicate significant differences among tissues (*p <* 0.05, Duncan’s multiple range test, *n* = 5).

The level of transcriptional expression of *CsPPO1* in the stem is significantly higher than that in the roots, leaves, and flowers (Figure 4A). The expression level of *CsPPO2* in the roots is significantly higher than that in the leaves, flowers, and stems, while the expression of *CsPPO2* in the stems is significantly lower than that in the leaves and flowers (Figure 4B).

### 2.5. Jasmonic Acid Elicits the Expression of CsPPO1 and CsPPO2 Differentially

**Figure 5:**
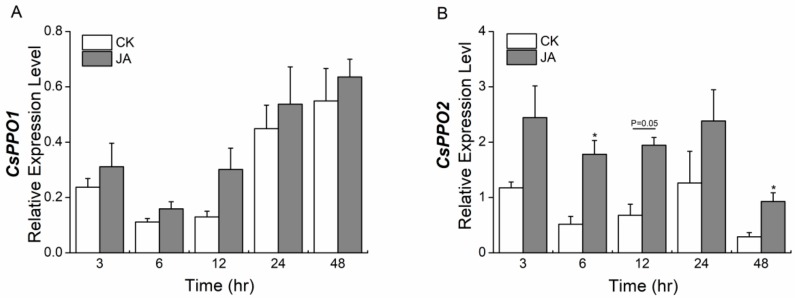
Mean levels of transcriptional expression (±SE) of (**A**) *CsPPO1* and (**B**) *CsPPO2* in tea leaves of jasmonic acid (JA)-treated plants and control plants. *CsGAPDH* was used as a reference gene. The asterisks indicate significant differences between treatments and controls (* *p* < 0.05, Student’s *t*-test, *n* = 5).

Exogenous application of JA significantly elicited the expression of *CsPPO2* at 6 and 48 h after the start of treatment, but not of *CsPPO1* (Figure 5).

### 2.6. Infestation of Caterpillars Elicit the Expression of CsPPO1 and CsPPO2

**Figure 6:**
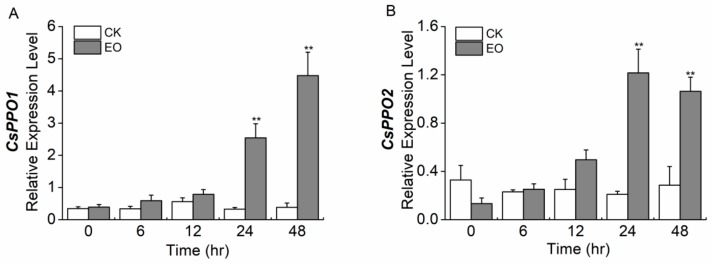
Mean levels of transcriptional expression (±SE) of (**A**) *CsPPO1* and (**B**) *CsPPO2* elicited in leaves of *Camellia sinensis* by the infestation of *Ectropis obliqua*. *CsGAPDH* was used as a reference gene. The asterisks indicate significant differences between treatments and controls (* *p* < 0.05, ** *p* < 0.01, Student’s *t*-test, *n* = 5).

The infestation of *E. obliqua* larvae significantly elicited the expression of *CsPPO1* and *CsPPO2* (Figure 6). Levels of *CsPPO1* and *CsPPO2* in caterpillar-infested tea plants at 24 and 48 h after the start of treatment were significantly higher than those in control plants: 7.8- and 11.6-fold higher, and 5.8- and 3.7-fold higher, respectively.

### 2.7. Regurgitant Elicits the Expression of CsPPO1 and CsPPO2 Differentially

**Figure 7:**
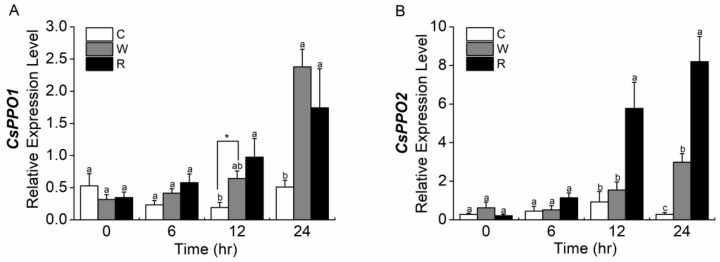
Mean levels of transcriptional expression (±SE) of (**A**) *CsPPO1* and (**B**) *CsPPO2* in treated tea leaves elicited by mechanical wounding supplemented with distilled water or regurgitant and intact control plants (C). *CsGAPDH* was used as a reference gene. For each time point, different letters indicate significant differences among treatments (*p* < 0.05, Duncan’s multiple range test, *n* = 5). The asterisks indicate significant differences between treatments and controls (* *p* < 0.05, Student’s *t*-test, *n* = 5).

Wounding plus regurgitant and wounding plus deionized water significantly elicited the expression of *CsPPO1* and *CsPPO2* (Figure 7). The expression of *CsPPO1* was significantly induced in plants by treatment involving wounding plus deionized water or wounding plus regurgitant at 12 h after the start of treatment, compared to the expression in intact plants, whereas the expression of *CsPPO2* was only significantly induced by wounding plus regurgitant at 12 h. Moreover, plants treated with wounding plus regurgitant significantly upregulated the expression of *CsPPO2* compared to plants treated with wounding plus deionized water at 12 and 24 h after the start of treatment (Figure 7B), approximately 2.22 and 3.73 times, respectively. But, the expression of *CsPPO1* did not differ significantly regardless of whether or not plants had been treated by wounding plus deionized water or wounding plus regurgitant at 12 and 24 h after the start of treatment (Figure 7A).

### 2.8. Separated Compounds of Regurgitant Elicit the Expression of CsPPO1 and CsPPO2 Differentially

**Figure 8:**
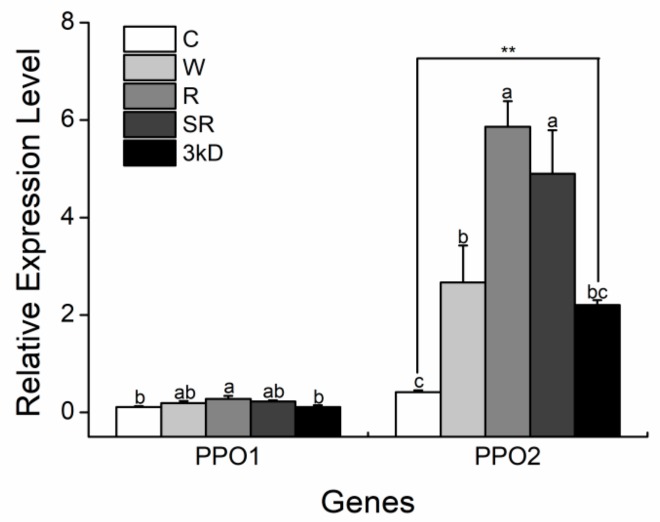
Mean levels of transcriptional expression (±SE) of *CsPPO1* and *CsPPO2* in treated tea leaves elicited by mechanical wounding supplemented with distilled water (W), diluted regurgitant (R), sterile extract (SR), sterile extract without 3-kDa molecules(3kD) and intact control plants (C). *CsGAPDH* was used as a reference gene. For each time point, different letters indicate significant differences among treatments (*p* < 0.05, Duncan’s multiple range test, *n* = 5). The asterisks indicate significant differences between treatments and controls, ** *p* < 0.01, Student’s *t*-test, *n* = 5).

The expression of *CsPPO1* was only significantly induced by the wounding plus diluted regurgitant treatment at 24 h after the start of treatment, compared to the expression in intact plants, whereas the expression of *CsPPO2* was significantly induced by all treatments at 24 h; in addition, the plants treated with wounding plus diluted regurgitant and sterile extract significantly upregulated the expression of *CsPPO2* compared to plants treated with wounding plus deionized water (Figure 8). Under treatment of the three separate compounds of regurgitant, the expressions of *CsPPO1* between wounding plus diluted regurgitant treatment and wounding plus sterile extract removed 3-kDa molecular weight off differ (Figure 8), whereas the expression levels of *CsPPO2* between wounding plus diluted regurgitant treatment and wounding plus sterile extract treatment are the same; but, the expression of both had significant differences with the treatment under wounding plus sterile extract without 3-kDa molecules (Figure 8).

## 3. Discussion

PPOs play multiple roles in *C. sinensis*, such as the elicitation of defenses against *E. obliqua* and the oxidation of flavanols to theaflavins and thearubigins during tea processing [4,38,39]. However, which members of the *CsPPO* gene family are involved in the defense response of tea plants have not yet been identified. In the present study, two new *PPO* genes, *CsPPO1* and *CsPPO2*, both of which had high similarity with *PPOs* from other plants, were obtained by rapid amplification of cDNA ends PCR (RACE-PCR) from leaves of *C. sinensis*. The transit peptides were predicted in the N-terminal region of CsPPO1 and CsPPO2, indicating that the tea plant’s PPO is a chloroplastic enzyme, which is consistent with the report of Halder et al. [40] and similar to other plant PPO proteins [41,42,43]. Moreover, the deduced amino-acid sequences of CsPPO1 and CsPPO2 were found to contain three conserved domains (Tyrosinase, PPO1-DWL, and PPO-KFDV), which are considered to be expression sequence tags of PPOs [44]. That CsPPO1 has only one copper-binding site and CsPPO2 contains two (Figure 1) suggests that they may have different functions, similar to *PPO* genes of strawberry (*Fragaria vesca*) [45]. Furthermore, results from 25 different plants involving 46 *PPO* genes in a phylogenetic analysis suggest that *CsPPOs* have a common ancestor with the red clover, lotus flower, and pokeweed, as these species were clustered on the adjoining branch (Figure 2). Results of phylogenetic and gene-structure analysis indicate that *PPO* genes are relatively conserved across different species. *SmePPO5*, *SmePPO6*, *SlyPPOF*, *PINPPO1*, *PINPPO2*, *PtdPPO1*, *PtdPPO3*, *pAPO5*, *CsPPO1*, and *CsPPO2* were wound-induced, while *SlyPPOF* and *PtdPPO3* were induced by pathogens. *AmAS1* has been unambiguously demonstrated to play a role in the biosynthesis of chalcone-derived yellow-colored aurone pigments in *Antirrhinum majus* (snapdragon) [46], and *LtLH* is an enantiospecific PPO involved in the biosynthesis of linked lignins that have been isolated and characterized in *Larrea tridentata* (creosote bush) [47,48,49]. Genes with functions similar to those of PPOs from different species do not cluster together in the phylogenetic analysis (Figure 2), suggesting that the adaptation of *PPO* genes for defense evolved independently in different plants. This result might explain the differences, which are consistent with those reported by Schmidt et al. [50]. Wu et al. expressed a reported *CsPPO* in *E. coli* and found that recombinant CsPPO appears as an inclusion body upon expression in *E. coli* despite the removal of chloroplast-targeting transit peptide; subsequently, they attempted to solubilize inclusion bodies in a suitable buffer; the specific activity of PPO was only 19.01 U/mg protein [51]. Our results show that the *CsPPO1* and *CsPPO2* genes highly express in *E. coli* cells, but the recombinant proteins exist in the form of an inclusion body (Figure 3), which are similar to the results of Liu et al. [29]. In a follow-up study, we need to solubilize inclusion bodies in a suitable buffer to measure the specific enzymatic activity of CsPPO1 and CsPPO2.

Previous studies have reported that different *PPO* gene members have distinct expression levels in various tissues [22,45,47,52]. For instance, although three *PPO* genes have been found in a poplar hydrid (*Populus trichocarpa × P. deltoides*), *PtdPPO1* is exclusively expressed in damaged leaves, whereas *PtdPPO2* and *PtdPPO3* are predominantly expressed in stems, petioles, or roots [52]. Here, we found that *CsPPO1* was highly expressed in stems, and *CsPPO2* was highly expressed in roots. In other plants’ *PPO* gene families, the expression patterns of some members are similarly organ-specific with *CsPPO1* and *CsPPO2*, such as *SmPPO1*, *SmPPO16*, *FaPPO4*, *PtrPPO2*, and *PtrPPO3*, which are highly expressed in roots, and *SmPPO11*, which is highly expressed in stems [22,45,52]. The diverse expression profiles identified for *PPO* genes in different tissues suggest that they may have diverse functional roles. For example, *AmAS1* from snapdragon is localized in vacuoles and specifically catalyzes the formation of aurones from chalcones [46]; Li et al. [22] reported that eight *SmPPOs* that were expressed in *S. miltiorrhiza* roots have potential in lithospermic acid B biosynthesis and metabolism. The roots of tea plants are a component of traditional Chinese medicine, and have been reported to have pharmacological effects [53]; *CsPPO2* was highly expressed in the roots, suggesting that it may be involved in the biosynthesis and metabolism of phenolic acid in roots—a hypothesis that needs to be verified. However, our study mainly focused on *CsPPOs* with defensive roles against leaf-feeding pests, so we paid attention to the expression of *CsPPOs* in leaves but not to that in roots. What the biological meaning of *CsPPO2* is, which was highly expressed in the roots but plays important role in defending against *E. obliqua*, needs to be investigated further.

Strong evidence has shown that PPOs play defensive roles in tomato, poplar (*Populus trichocarpa*), strawberry, and some other plants [12,13,18,45,52,54,55,56]. Among the 15 members of the poplar *PPO* gene family, levels of transcriptional expression of *PtrPPO1*, *PtrPPO3*, and *PtrPPO11* were significantly induced by mechanical damage, the exogenous application of MeJA, or the infection of *Melampsora laricipopulina*, while others are developmentally regulated [48,57]. Similar results have also been reported in tomato, apple (*Malus domestica*), and pineapple (*Ananas comosus*) [58,59,60]. Our current results showed that the expression of *CsPPO1* and *CsPPO2* was wound- and *E. obliqua*-induced (Figure 6A,B), indicating that both were probably promoted to play defensive roles in *C. sinensis*. In contrast, JA treatment induced only the expression of *CsPPO2*, not of *CsPPO1* (Figure 5). The situation is similar in poplar [48], apple [58], and pineapple [59], where *PtrPPO5*, *pAPO5*, *PINPPO1*, and *PINPPO2* were found to be the only wound-induced *PPOs*. In other plants, most *PPO* genes are not wound- or herbivore-induced [48]. Nevertheless, the infestation of herbivores not only mechanically damaged plants but also introduced OS into the wounding sites [32,36,61]. Musser et al. [36] found that the caterpillar labial saliva of cotton bollworm (*Helicoverpa zea*) alters gene expression in the tomato plant. Our results showed that regurgitant could upregulate the expression level of *CsPPO2* but not of *CsPPO1* more significantly than treating the plant with wounding plus deionized water at 12 and 24 h after the start of the experiment (Figure 7). This result indicates that the accumulation of *CsPPO2* can be upregulated by regurgitant and may constitute a more meaningful defense against *E. obliqua* than the accumulation of *CsPPO1*. The disparity between the transcripts of *CsPPO1* and of *CsPPO2* may have resulted from their different structural attributes, as stated above. Similar disparities were previously found in other plants as well [22,57,59]. 

Several examples have shown that the components of OS can interfere with, or even suppress, the activation of defensive responses in plants [62]. Among the known herbivore-associated molecular patterns (HAMPs) [63], fatty acid–amino acid conjugates are widely distributed in the OS of lepidopteran insects and elicit specific responses in various plants; furthermore, H_2_O_2_, which is produced by glucoseoxidase in OS, is also believed to take part in activating insect feeding-induced defensive reactions [64]. In the present study, we found that *CsPPO2* was significantly amplified by diluted regurgitant, but its expression level did not differ significantly between wounding plus sterile extract and wounding plus diluted regurgitant, suggesting the active component(s) was not microbial (Figure 8). Chung et al. [32] found the Colorado potato beetles (CPB, *Leptinotarsa decemlineata*) can exploit orally secreted bacteria to suppress plant defenses, and Wang et al. (2016) found different microbes in insects can have species-specific effects on different host plants. The microbes in the regurgitant of *E. obliqua* may have effects on other defense genes that need to be verified. Treatments of wounding plus diluted regurgitant and sterile extract significantly elicited the expression of *CsPPO2* compared with its expression in wounding plus deionized water, and wounding plus sterile extract removed small molecules off and intact plants (Figure 8), which suggests the active component(s) of the regurgitant that elicited the expression of *CsPPO2* may contain in small molecules (within 3-kDa molecular weight). Similarly, the difference in responses between the expression of *CsPPO1* under wounding plus diluted regurgitant and wounding plus sterile extract without small molecules supported this result (Figure 8). 

Previously, our group noted the infestation of *E. obliqua* or wounding plus the regurgitant significantly suppressed wound-induced PPO activities [4]. Given our earlier results and our results here, we hypothesize that there are other *PPO* gene members that are particularly responsive to the infestation of *E. obliqua* in the tea plant; the assumption needs to be further investigated. Meanwhile, microRNAs (miRNAs) represent key post-transcriptional regulators of eukaryotic gene expression and play important roles in stress responses. The prediction of miRNAs in *CsPPO1* and *CsPPO2* indicates that miRNAs are involved the post-transcriptional regulation of *PPOs*, which may lead to the difference between our earlier results and results here. 

The regurgitant from *E. obliqua* was separated into its components for the first time in this study, and we found the active component(s) may elicit the expression of *CsPPO2* contained in small molecules (within 3-kD molecular weight). Future studies are planned identifying the active components in regurgitant that launch the induced defense response of the tea plant against this herbivore.

## 4. Materials and Methods

### 4.1. Insects

*E. obliqua* eggs were originally obtained from Plantation Centre of Tea Research Institute of Chinese Academy of Agricultural Sciences (CAAS), Hangzhou, Zhejiang, and maintained in an insectary. The newly hatched larvae were raised in net cages (75 × 75 × 75 cm) with potted fresh tea shoots and kept in a controlled climate room programmed at 26 ± 2 °C, 70 ± 5% RH (relative humidity), and 12-h photophase. After one generation, *E. obliqua* caterpillars were used for the experiments.

### 4.2. Regurgitant Collection and Separation

Regurgitant was collected from the oral cavity of 4th-instar *E. obliqua* with a P200 Pipetteman (Gilson, Middleton, WI, USA). The collected regurgitant was centrifuged for 5 min at 10,000× *g*, after which the supernatant was collected and stored at −80 °C for leaf treatment. Preliminary separation of the regurgitant was carried out according to the method described in Ray et al. [65] (2015) with minor revision. The collected regurgitant was diluted 1:4 *v*/*v* with water and filtered through Miracloth (EMD Millipore, Billerica, MA, USA) to remove debris. Then, the diluted regurgitant was sterilized in 0.2-μm filters (EMD Millipore), and the sterile regurgitant was concentrated using centrifugal columns with a 3-kDa molecular weight cut-off (Pall Life Sciences, Louisville, KY, USA) to remove small molecules present in the extract.

### 4.3. Tea Plants and Treatments

One-year-old Longjing 43 tea plants were planted individually in plastic pots (14 cm diameter × 15 cm high) and grown in a greenhouse (26 ± 2 °C, relative humidity of 70–80%, 12 h photophase), irrigated once every other day and fertilized with rapeseed cake once a month. The light intensity for plants was about 450 μmol·m^−2^·s^−1^ during the photophase. Three-year-old plants were used for experiments. Potted plants were washed under running water and then air-dried. Samples and plant treatments were prepared as follows:

#### 4.3.1. Different Tissues

**Figure 9:**
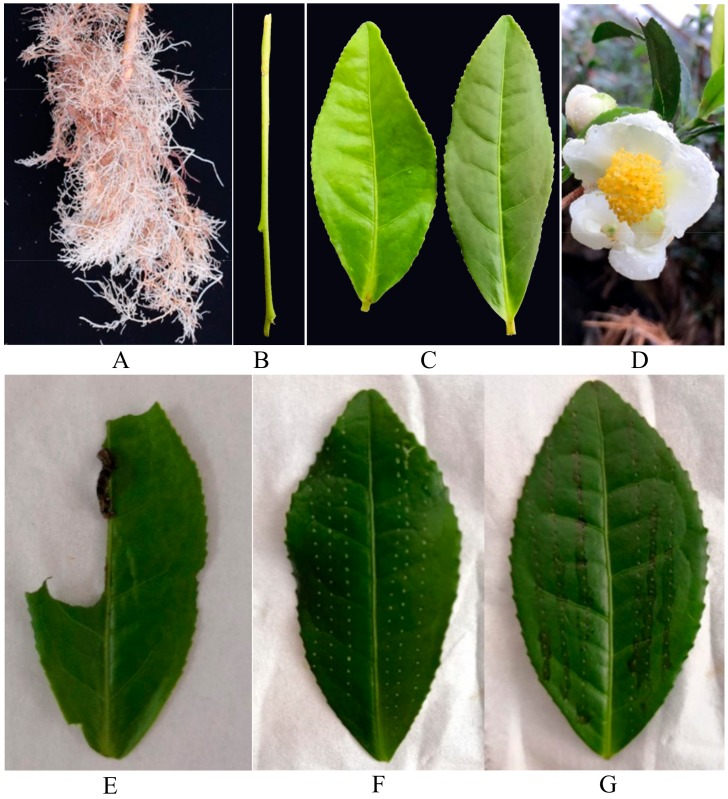
Characteristic photographs of treatments. (**A**): Root. (**B**): Stem. (**C**): Leaf. (**D**): Flower. (**E**): Caterpillar infestation. (**F**): Mechanical damage with deionized water. (**G**): Mechanical damage with regurgitant.

Different tissues, including roots (Figure 9A), tender internodes between the fourth to seventh (Figure 9B), leaves (the second to third intact leaves away from the terminal growing point, Figure 9C) and half-open flowers (Figure 9D) were harvested from the same plant, immediately frozen in liquid nitrogen and then stored at −80 °C. Five replications were carried out.

#### 4.3.2. JA Treatment

JA (Sigma Chemical Co., St. Louis, MO, USA) was dissolved in a small amount of ethanol and made up to a concentration of 150 μg/mL in 50 mM sodium-phosphate buffer (titrated with 1 M citric acid until pH 8). Plants were individually sprayed with 8 mL of JA solution. Control plants were individually sprayed with 8 mL of the buffer. Plants were treated at 9 am and then placed in a controlled-climate room that was maintained at 26 ± 2 °C, 12-h photophase, and 80% RH. The second leaves were harvested at 0, 3, 6, 12, 24, and 48 h after the start of treatment. Five replications were carried out.

#### 4.3.3. Caterpillar Infestation

The second leaf of each plant was covered with a fine-mesh sleeve into which 2 3rd-instar caterpillars that had been starved for 10 h were introduced. Plants with only their second leaves covered with fine-mesh sleeves were used as controls. The second leaves were harvested at 0, 6, 12, 24, and 48 h after the start of treatment (Figure 9E). Five replications were carried out.

#### 4.3.4. Regurgitant Induction

The mechanical damage was made by a fabric pattern wheel following the method described in Lou and Baldwin [66]. Each leaf was rolled 6 times, and 15 μL whole regurgitant from 4th-instar larvae was added to the puncture wounds on each leaf (Figure 9G). 15 μL of deionized water was added to the damaged leaves for wounding treatment (Figure 9F). Intact second leaves were used as controls. All the second leaves were harvested at 0, 6, 12, and 24 h after the start of treatment. Five replications were carried out.

#### 4.3.5. Separated Regurgitant Induction

15 μL of deionized water, diluted regurgitant, sterile extract, and sterile extract without 3 kDa molecular weight were added to damaged leaves. Intact second leaves were used as controls. Second leaves were harvested at 24 h after the start of treatment. Five replications were carried out.

### 4.4. RNA Extraction and cDNA Synthesis

Total RNA was isolated with TRIzol™ kit, according to the manufacturer’s instructions (TIANGEN, Beijing, China). Quality and concentration were checked by agarose-gel electrophoresis and spectrophotometer analysis, and the RNA was stored at −80 °C until use. First-strand cDNA was synthesized from total RNA using a PrimerScript^®^ RT Reagent Kit (Takara, Dalian, China) according to the manufacturer’s instructions. After reverse transcription, the synthesized cDNA was stored at −20 °C for future use.

### 4.5. Cloning Full-Length CsPPO Gene Sequences

The cDNA fragments were obtained from the previous transcriptome of Longjing 43. The 5′ and 3′ sequences of *CsPPO1* and *CsPPO2* were acquired by rapid amplification of RACE-PCR using the manufacturer’s protocol (SMARTer^®^ RACE 5′/3′Kit, Clontech Lab, Inc., Mountain View, CA, USA). The PCR products were ligated into a pMD-19T easy vector (Takara, Dalian, China) after purification. After the 3′ and 5′ sequences were obtained, the full-length cDNA sequences of *CsPPO1* and *CsPPO2* were cloned by RT-PCR reaction using primers PPO1-full1, PPO1-full2, PPO2-full1, and PPO2-full2. All the primers used in RACE are listed in Table 2.

### 4.6. Bioinformatic and Phylogenetic Analysis

The BLAST program at the NCBI Web site (https://blast.ncbi.nlm.nih.gov/Blast.cgi) was used to compare the amino-acid sequences of CsPPO1 and CsPPO2. The ORF was analyzed using the ORF finder (https://www.ncbi.nlm.nih.gov/orffinder/). The molecular weight (Mw) and theoretical isoelectric (pI) points of *CsPPO1*/*CsPPO2* were calculated using the online computer pI/Mw tool (http://cn.expasy.org/tools). The putative domains were identified using the InterPro database (http://www.ebi.ac.uk/interpro/), and the Cu-binding sites were analyzed with the website (http://www.expasy.ch/prosite). ClustalW2 software (Lynnon Biosoft, Los Angeles, CA, USA) was used to align the CsPPO1 and CsPPO2 sequences with other PPO sequences from related species. The phylogenetic tree was constructed by MEGA6 (http://www.megasoftware.net/) using the neighbor-joining method with the bootstrap values calculated from 1000 replicates.

### 4.7. Recombinant Protein Expression in Escherichia coli

Plasmid vector pGEX-4T-2 was used to produce recombinant protein. The plasmids pTA-CsPPO1 and pTA-CsPPO2 were cloned by the forward primers with *BamH I* site and the reverse primers with *Sma I* site (Table 2). The pGEX-4T-2 vector, plasmid pTA-CsPPO1, and pTA-CsPPO2 were digested with the restriction enzymes *BamH I* and *Sma I* and ligated to obtain the expression plasmid pGEX-4T-2/CsPPO1 and pGEX-4T-2/CsPPO2. After being sequenced to confirm the cloned fragments, the constructs were transformed into the *E. coli* BL21 (DE3). Cells were grown at 37 °C overnight in LB media containing ampicillin (50 μg·mL^−1^). Following centrifugation, *E. coli* cells were adjusted to OD_600_ = 0.5–0.8, and the production of recombinant protein was induced by adding 1 mM isopropyl-β-d-thiogalactopyranoside (IPTG) to a final concentration of 0.2 mM. No IPTG induction samples were treated as controls. Incubation was continued for 6 h at 18 °C and finally analyzed by sodium dodecylsulfate-polyacrylamide gel electrophoresis (SDS-PAGE).

### 4.8. Real-Time PCR (qPCR) Analysis

RT–qPCR was carried out to investigate the expression profiles of *CsPPO1* and *CsPPO2* in different tissues and under different treatments. Five independent biological samples were used, and the cDNA from different tissues or different treatments was used as a template. The *GAPDH* (Accession No. GE651107, Deng et al., 2016) was used as the internal standard. The RT-qPCR primers were designed by primer premier 5.0 software (PREMIER Biosoft, Palo Alto, CA, USA), and all the primers are listed in Table 1. The RT-qPCR was performed on a LightCycler 480 system (Roche Diagnostics, Mannheim, Germany) using a Premix Ex Taq kit (TaKaRa, Dalian, China) with a 20 μL reaction mixture containing 1 μL cDNA, 10 μL qPCR Master Mix (Mountain View, CA, USA), 1 μL of each specific primer (0.2 mM), and 7 μL nuclease-free water. The qPCR program included a preliminary step at 95 °C for 30 s, 40 cycles of a denaturation at 95 °C for 10 s, and an annealing and extension step at 58 °C for 1 min. The relative expressions were calculated by 2^−^^ΔΔ*C*t^ method.

### 4.9. Statistical Analysis

All statistical analyses were performed by using the Statistica (Statistica, SAS, Institute Inc., Cary, NC, USA). The differences among the transcriptional levels of *CsPPO1* and *CsPPO2* expressed in different tissues, and among tissue from intact plants, and from plants subjected to wounding and regurgitant induction were analyzed by using one-way ANOVAs with *p* < 0.05 indicating statistical significance. Differences in the levels of gene expression between *E. obliqua-*infested and intact tea plants, and JA-treated and intact tea plants were determined by Student’s *t*-test.

## Figures and Tables

**Table 1:** Percentage of similarity among three tea-plant PPO sequences, calculated for both nucleotide and amino-acid sequences using MEGALIGN (DNAStar). Protein identities are in bold.

Name	CsPPO1	CsPPO2	CsPPO
CsPPO1	100.0	**68.45**	**69.83**
CsPPO2	71.68	100.0	**71.14**
CsPPO	73.27	75.27	100.0

**Table 2:** Primers used for cloning and analysis of *CsPP1* and *CsPPO2*.

Primers	Purpose	Primer Sequence (5′-3′)
GSP1-3	3′-RACE	AATGTGGATCGGATGTGG
NGSP1-3	3′-RACE	CAGAGACCGAAGAAATCAAG
GSP2-3	3′-RACE	GGTTTGTGTTCTATGATGAG
NGSP2-3	3′-RACE	AGAAGGATGATGAAGAGGAG
GSP1-5	5′-RACE	GAGCCATGAGTTGTGGACTTGAAG
NGSP1-5	5′-RACE	TGGTGATAAGCTCCGTCGCAATAA
GSP2-5	5′-RACE	ATCCGACCCGTTGTAATCCA
NGSP2-5	5′-RACE	CGAAGAAGTGGAGGTAGTAT
UPM	RACE	Long primer: CTAATACGACTCACTATAGGGCAAGCAGTGGTATCAACGCAGAGT
		Short primer: CTAATACGACTCACTATAGGGC
PPO1-full1	Clone	ATGAATTCTCTTCCACCATCA
PPO1-full2	Clone	TTAGGAATCAAACTCAATCTTG
PPO2-full1	Clone	ATGGCTTCTTTTCCACCTTC
PPO2-full2	Clone	TCAAGAATCAAACTCTATCTTGA
PPO1-PF	protein	CTGGTTCCGCGTGGATCCATGAATTCTCTTCCACCATCATGCA
PPO1-PR	protein	CGCTCGAGTCGACCCGGGTTAGGAATCAAACTCAATCTTG
PPO2-PF	protein	CTGGTTCCGCGTGGATCCATGGCTTCTTTTCCACCTTC
PPO2-PR	protein	CGCTCGAGTCGACCCGGGTCAAGAATCAAACTCTATCTTGA
GAPDH-F	QPCR	GACTGGAGAGGTGGAAGAGC
GAPDH-R	QPCR	AGCCATTCCAGTCAATTTCC
PPO1-RTF	QPCR	CCATCTGGAAGAGTTTGGGT
PPO1-RTR	QPCR	CCTTCACTTTGACAGGCTGA
PPO2-RTF	QPCR	CGGAATGCCAATGCCTGCAA
PPO2-RTR	QPCR	AGTTGGATCCGACCCGTTGT

## Abbreviations

PPOs	Polyphenol oxidases
ORF	Open reading frame
JA	Jasmonic acid
MeJA	Methyl jasmonate
SA	Salicylic acid
ET	Ethylene
OS	Oral secretions
HAMPs	Herbivore-associated molecular patterns
CPB	Colorado potato beetles
CAAS	Chinese Academy of Agricultural Sciences
Mw	Molecular weight
pI	Isoelectric point
IPTG	Isopropyl-β-d-thiogalactopyranoside
SDS–PAGE	Sodium dodecylsulfate-polyacrylamide gel electrophoresis
